# Serum GDF15 as a potential biomarker for treatment response and survival in patients with resectable locally advanced gastric cancer undergoing neoadjuvant chemotherapy

**DOI:** 10.3389/fmolb.2026.1948454

**Published:** 2026-10-07

**Authors:** Can Cao, Shunda Wang, Shikang Ding, Hongyun Huang, Tian Yu, Moxi Chen, Yibin Xie, Jianchun Yu

**Affiliations:** 1 Department of General Surgery, Peking Union Medical College Hospital, Chinese Academy of Medical Sciences and Peking Union Medical College, Beijing, China; 2 Department of Pancreatic and Gastric Surgery, National Cancer Center/ National Clinical Research Center for Cancer/ Cancer Hospital, Chinese Academy of Medical Sciences and Peking Union Medical College, Beijing, China

**Keywords:** growth differentiation factor 15, locally advanced gastric cancer, neoadjuvant chemotherapy, survival prognosis, treatment response

## Abstract

**Background:**

Patients with locally advanced gastric cancer (LAGC) typically require neoadjuvant chemotherapy (NAC) to reduce tumor burden and improve surgical resectability. However, treatment responses to NAC vary substantially among individuals. This study investigated the potential value of baseline serum growth differentiation factor 15 (GDF15) for identifying patients at risk of poor treatment response and unfavorable survival outcomes among patients with LAGC receiving NAC.

**Methods:**

We retrospectively identified 318 eligible patients with LAGC who received NAC and enrolled 30 healthy controls. Serum GDF15 levels were measured by ELISA. In the unmatched cohort, the median baseline serum GDF15 concentration (2,147.3 pg/mL), determined before PSM, was used as a fixed cutoff to define the GDF15-L and GDF15-H groups (n = 159 each). After 1:1 PSM, 256 patients (n = 128 per group) were included in the matched analyses. Comparative analyses included baseline characteristics, CT images, preoperative endoscopic biopsy specimens and postoperative pathological features. Survival outcomes (OS and DFS) were assessed via Kaplan-Meier analysis. Multivariate Cox regression identified prognostic factors, and a prognostic nomogram was constructed.

**Results:**

High GDF15 expression correlated with advanced age (P = 0.045), poor tumor differentiation (P = 0.040), and elevated serum CA72-4 (P = 0.028). The GDF15-L group demonstrated superior median OS (58.1 vs. 54.0 months, P = 0.031) and DFS (47.1 vs. 26.9 months, P = 0.002) compared to GDF15-H counterparts. Preoperative endoscopic biopsy demonstrated a positive correlation between tissue GDF15 expression and serum GDF15 levels, while post-NAC imaging indicated greater tumor regression in the GDF15-L group. The GDF15-L group exhibited a higher pathological complete response (pCR) rate in postoperative pathological features (P < 0.001) while the GDF15-H group exhibited increased positive lymph nodes (P = 0.018) and a higher incidence of newly detected distant metastasis during or after NAC (P = 0.024). Multivariate analysis identified high GDF15 expression (P = 0.021) and high CAP degree (P = 0.022) as independent risk factors for OS, with younger age being protective (P = 0.016). Based on these factors, a prognostic nomogram was constructed to predict 1-year, 3-year, and 5-year OS and DFS.

**Conclusion:**

Low baseline serum GDF15 levels were associated with favorable radiological and pathological responses and prolonged survival in patients with LAGC receiving NAC. Baseline serum GDF15 may help identify patients at increased risk of poor response and unfavorable prognosis and may therefore serve as a potential biomarker for pretreatment risk stratification. However, because all patients received NAC, these findings do not establish that GDF15 predicts differential benefit from NAC, and prospective validation is required.

## Introduction

Gastric cancer (GC) ranks as the fifth most prevalent malignancy and the fifth leading cause of cancer-related mortality globally (Bray et al., 2024). Patients with locally advanced gastric cancer (LAGC) constitute a substantial proportion of the overall GC cases, and the clinical manifestations and prognostic characteristics differ markedly from those of early-stage disease. Early-stage GC usually lack typical symptoms, leading to delayed diagnosis and aggressive progression, with most patients presenting at advanced stages associated with elevated therapeutic risks and dismal survival rates (Arnold et al., 2020). Although surgical resection remains the cornerstone of potentially curative treatment for LAGC, high rates of locoregional recurrence and distant metastasis persist even after radical resection with lymphadenectomy (Maehara et al., 2000; Rivera et al., 2007). Since the landmark MAGIC trial, neoadjuvant chemotherapy (NAC) has been proven to be beneficial in reducing tumor burden and metastatic lymph node involvement, thereby improving surgical feasibility and survival outcomes in LAGC patients (Cunningham et al., 2006). Subsequent trials, including FLOT4, PRODIGY, and RESONANCE-II, further validated survival benefits by comparing and optimizing NAC regimens (Al-Batran et al., 2019; Kang et al., 2021; Kang et al., 2024; Wang X. et al., 2021).

Despite these advances in the comprehensive treatment of LAGC, clinical outcomes remain suboptimal, as evidenced by the EORTC 40954, CRITICS, and JCOG0501 trials, which revealed heterogeneous responses to NAC, with a subset of patients experiencing treatment resistance or disease progression (Cats et al., 2018; Schuhmacher et al., 2010; Iwasaki et al., 2021). Consequently, identifying robust biomarkers to stratify NAC-responsive populations represents a critical unmet need in precision oncology. Growth differentiation factor 15 (GDF15), initially termed macrophage inhibitory cytokine-1 (MIC-1), belongs to the transforming growth factor-β (TGF-β) superfamily (Bootcov et al., 1997). Emerging researches highlight its upregulation in diverse pathological states, including malignancies, frailty, aging, chronic diseases, and cachexia (Corre et al., 2013; Myojin et al., 2021; Gonçalves et al., 2022; Conte et al., 2022; Groarke et al., 2024). Notably, elevated GDF15 expression has been implicated in aggressive tumor biology and unfavorable prognoses across multiple cancers (Zhu et al., 2024; Zabransky et al., 2024). However, systematic investigations and in-depth explorations into its role in predicting treatment response and survival outcomes of NAC in LAGC remain lacking.

Therefore, we conducted this retrospective study to evaluate whether baseline serum GDF15 could serve as a pretreatment risk stratification biomarker in LAGC patients receiving NAC, by analyzing its associations with radiological/pathological response and survival outcomes. We hypothesized that higher GDF15 levels would identify patients at greater risk of poor response and unfavorable prognosis, potentially enabling early stratification of NAC-responsive cohorts and guiding personalized treatment.

## Materials and methods

### Patient selection and data collection

We retrospectively analyzed 451 patients with gastric cancer histologically confirmed by endoscopic biopsy from a prospectively collected database at Peking Union Medical College Hospital between October 2016 and October 2022. Pretreatment clinical staging was determined according to the AJCC/UICC eighth-edition TNM classification on the basis of contrast-enhanced CT and the other staging examinations documented in the medical records. Inclusion criteria were: (1) locally advanced disease with a pretreatment clinical stage of cT2–cT4b, cN0–cN3, and cM0 (Amin et al., 2017); (2) receipt of NAC followed by surgery. All eligible patients had no evidence of distant metastasis at the initial pretreatment assessment. According to the AJCC/UICC eighth-edition clinical stage grouping, cT4b disease with any cN category and cM0 is classified as clinical stage IVA and was therefore eligible for inclusion. Exclusion criteria included: incomplete baseline/follow-up data (n = 38); clinical stage I disease treated with endoscopic mucosal resection (EMR)/endoscopic submucosal dissection (ESD) (n = 17); pretreatment clinical stage IVB disease with distant metastasis (any cT, any cN, and cM1; n = 30); unavailable preoperative serum or tissue samples (n = 32); squamous cell carcinoma histology (n = 5); and NAC discontinuation due to adverse effects or neoadjuvant radiotherapy (n = 11). Ultimately, 318 LAGC patients were included and blood samples were collected before NAC. Baseline serum GDF15 concentrations were measured in all 318 eligible patients before PSM. Patients were subsequently dichotomized according to the median serum GDF15 concentration determined in this unmatched cohort, yielding 159 patients in each GDF15 group. Thirty age-matched healthy controls (median age: 59.5 years, interquartile range [IQR]: 52–66) were additionally enrolled (Figure 1A).

**FIGURE 1:**
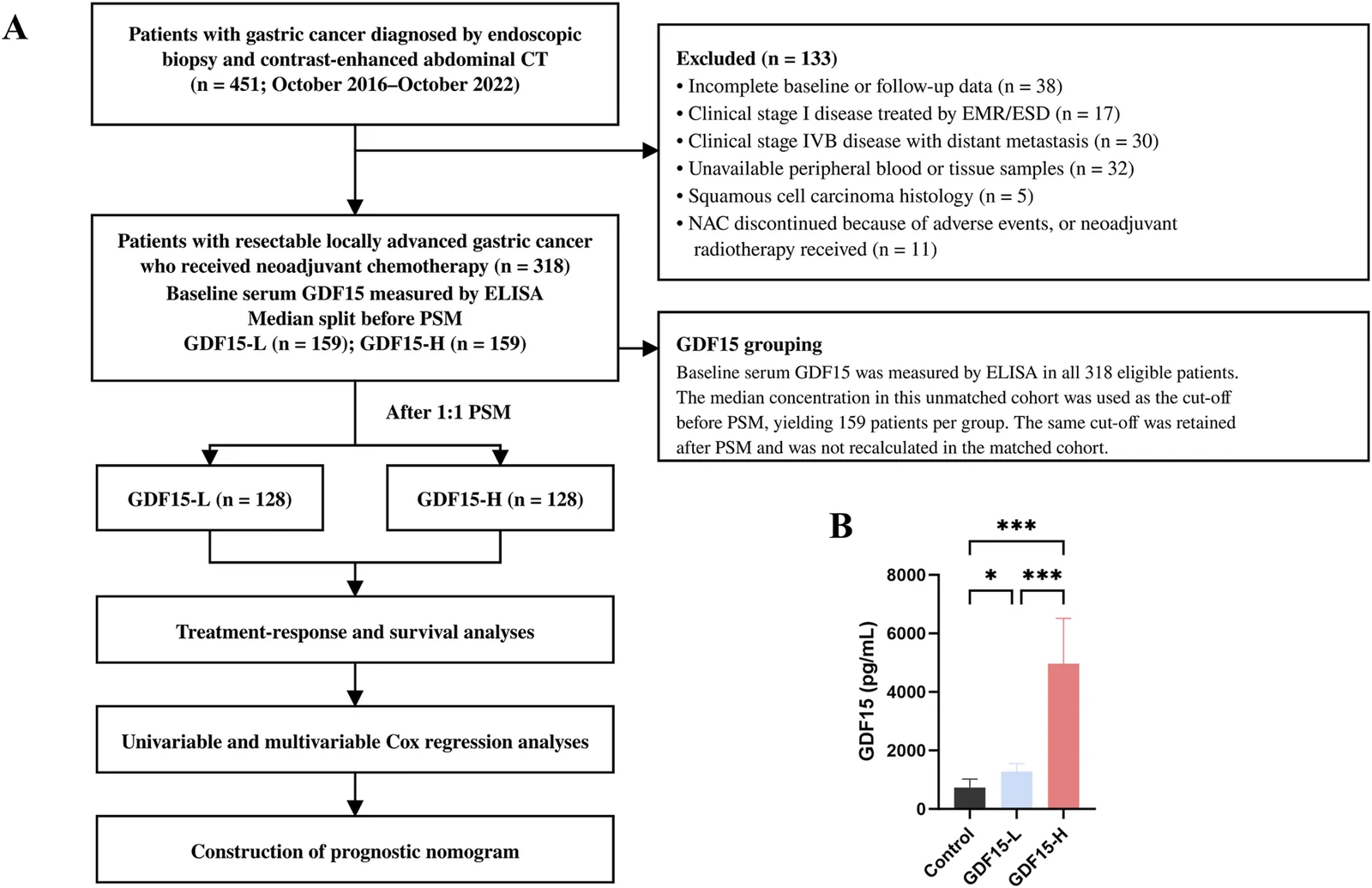
Flowchart of the study and serum GDF15 grouping. **(A)** Study flowchart. After application of the eligibility criteria, 318 patients underwent baseline serum GDF15 measurement. The median concentration in this unmatched cohort was used as the cutoff before PSM, resulting in 159 patients in each GDF15 group. Following 1:1 PSM, 128 patients were retained in each group. **(B)** Serum GDF15 concentrations in healthy controls and patients in the matched GDF15-L and GDF15-H groups. The groups were defined using the fixed pre-PSM median cutoff of 2,147.3 pg/mL. *p < 0.05 and ***p < 0.001.

Demographic and clinicopathological variables were collected, including sex, age, Nutritional Risk Screening (NRS) 2002 score, tumor location, differentiation grade, Lauren classification, signet-ring cell features, HER-2 status, NAC regimen/cycles, pretreatment clinical cT/cN/cTNM stage, and serum levels of GDF15, CEA, CA19-9, and CA72-4. All patients underwent gastrectomy with D2 lymphadenectomy achieving R0 resection. Post-NAC clinicopathological features included vascular invasion, perineural invasion, ypT stage, ypN stage, ypTNM stage, and College of American Pathologists tumor regression grade (Burghart and Jain, 2021). All patients were classified as cM0 at baseline. Distant metastasis that was absent on baseline staging but first detected between the initiation of NAC and surgery was recorded separately as “newly detected distant metastasis during or after NAC.” Distant metastasis was defined as metastasis to a distant organ, peritoneal dissemination, or involvement of nonregional lymph nodes, as determined by post-NAC imaging, intraoperative findings, and/or histopathological examination.

Patients underwent a standardized postoperative surveillance program, with follow-up visits every 3 months during the first postoperative year, every 6 months during years 2–3, and annually thereafter. Follow-up data were obtained through structured telephone interviews and review of electronic medical records. Dates of tumor recurrence, metastasis, death, and last follow-up were recorded. The primary endpoints were overall survival (OS), defined as the interval from initiation of NAC to death from any cause, and disease-free survival (DFS), defined as the interval from initiation of NAC to tumor recurrence or death from any cause, whichever occurred first. Patients without an event were censored at the date of their last follow-up. The last follow-up was conducted in June 2024.

For focused molecular validation, 24 patients with available frozen pretreatment endoscopic biopsy specimens and complete baseline serum GDF15 and postoperative CAP grading data were selected from the matched cohort. The subset comprised six patients in each of the GDF15-L/CAP 0, GDF15-L/CAP 3, GDF15-H/CAP 0, and GDF15-H/CAP 3 subgroups. Eligible patients were randomly selected within each subgroup. CAP grades 0 and 3 were selected because they represented the most contrasting pathological response phenotypes after NAC; patients with CAP grades 1 or 2 were not included in this validation analysis.

The study protocol was approved by the Institutional Review Board of Peking Union Medical College Hospital and conducted in accordance with the Declaration of Helsinki. Written informed consent was obtained from all participants or their legal guardians.

### Neoadjuvant chemotherapy regimen

NAC regimens (SOX, XELOX, or FOLFOX) followed NCCN and ESMO guidelines (Ajani et al., 2013; Van Cutsem et al., 2011). The neoadjuvant chemotherapy (NAC) regimens for patients included SOX, XELOX, and FOLFOX. The SOX regimen consisted of S-1 and oxaliplatin. The XELOX regimen comprised capecitabine and oxaliplatin. The FOLFOX regimen included 5-fluorouracil, leucovorin, and oxaliplatin. All regimens were administered in 3-week cycles for 1–4 cycles.

### Enzyme-linked immunosorbent assay (ELISA)

Serum GDF15 concentrations were quantified using a commercial Human GDF-15 Sandwich ELISA Kit (#KE00108, Proteintech, Rosemont, IL, United States) according to the manufacturer’s instructions. The assay was performed using the GDF15 capture antibody-precoated 96-well microplate (8-well × 12 strips) supplied with the kit. The detection range of the assay was 1,250–40,000 pg/mL, with a sensitivity of 300 pg/mL. Serum samples were diluted 1:4 with Sample Diluent PT1, and 100 μL of each diluted serum sample, was added to each well. Standards and diluted serum samples were analyzed in duplicate (two technical replicates per sample). The plate was sealed and incubated at 37 °C for 2 h, followed by four washes with 1× wash buffer. Subsequently, 100 μL of HRP-conjugated detection antibody solution was added to each well, and the plate was incubated at 37 °C for 40 min. After four additional washes, 100 μL of tetramethylbenzidine (TMB) substrate solution was added to each well, followed by incubation at 37 °C for 15–20 min in the dark. The reaction was terminated by adding 100 μL of stop solution to each well.

Absorbance at 450 nm was measured using a microplate reader (BioTek Synergy H1) immediately after addition of the stop solution and within 5 min. A standard curve was generated for each assay using the GDF15 standards, and serum GDF15 concentrations were calculated using a four-parameter logistic (4-PL) regression model. For diluted samples, the concentrations obtained from the standard curve were multiplied by the corresponding dilution factor. The mean value of the duplicate measurements was used for subsequent statistical analyses.

For GDF15 grouping, the median baseline serum GDF15 concentration in the entire unmatched cohort (n = 318) was 2,147.3 pg/mL and was determined before PSM. This study-specific median was used as a fixed cutoff to define the GDF15-L (<2,147.3 pg/mL; n = 159) and GDF15-H (≥2,147.3 pg/mL; n = 159) groups. The cutoff was retained after PSM and was not recalculated in the matched cohort.

### Immunohistochemical (IHC) staining of pretreatment endoscopic biopsy specimens

Formalin-fixed, paraffin-embedded tumor sections (4 μm) underwent antigen retrieval using citrate buffer (pH 6.0), blocking with 5% BSA. The sections were incubated overnight at 4 °C with a rabbit monoclonal anti-GDF15 antibody (1:100 dilution; clone EPR19939; catalog No. ab206414; Abcam, Cambridge, United Kingdom). After washing, the sections were incubated with HRP-conjugated goat anti-rabbit IgG (H + L) secondary antibody (catalog No. GB23303; Servicebio, Wuhan, China). Immunoreactivity was subsequently visualized using a 3,3′-diaminobenzidine (DAB) chromogenic kit (catalog No. G1212-200T; Servicebio, Wuhan, China) according to the manufacturer’s instructions, followed by hematoxylin counterstaining. The slides were examined by two pathologists blinded to the clinical information. IHC scoring criteria included staining intensity (0, no staining; 1, weak; 2, moderate; 3, strong) and a semi-quantitative assessment of the percentage of positive cells (0, ≤5%; 1, 6%–25%; 2, 26%–50%; 3, 51%–75%; 4, 76%–100%). The final score was calculated by multiplying these two values (0–12). Based on the final score, patients were categorized into four subgroups: negative (0–2), weak (3–4), moderate (5–8), and strong (9–12).

### Quantitative real-time PCR analysis of pretreatment endoscopic biopsy specimens

For tissue-level validation, pretreatment endoscopic biopsy specimens from 24 patients were analyzed, including six patients in each of the GDF15-L/CAP 0, GDF15-L/CAP 3, GDF15-H/CAP 0, and GDF15-H/CAP 3 subgroups. The specimens were immediately frozen after collection and stored at −80 °C until analysis.

Total RNA was extracted using RNAiso Plus (Cat. No. 9108; Takara Bio, Shiga, Japan). RNA concentration and purity were assessed using a NanoDrop 2000 spectrophotometer (Thermo Fisher Scientific, Waltham, MA, United States). One microgram of total RNA was reverse-transcribed into cDNA using the PrimeScript RT reagent Kit with gDNA Eraser (Perfect Real Time; Cat. No. RR047A; Takara Bio). The resulting cDNA was diluted 20-fold before amplification.

Quantitative real-time PCR was performed in a total reaction volume of 20 μL using the HyperScript SYBR Green RT-qPCR Kit (Cat. No. Q301-01; Xinbei Biotechnology, Shanghai, China) on a QuantStudio7pro (Applied Biosystems, United States). The cycling conditions were 95 °C for 30 s, followed by 40 cycles of 95 °C for 5 s and 60 °C for 30 s. Melting-curve analysis was subsequently performed. Each sample was analyzed in three technical replicates, and the mean cycle-threshold value was used for analysis.

The primer sequences were as follows (5′–3′): GDF15 forward, CCCTGCAGTCCGGATACTC; GDF15 reverse, GAACAGAGCCCGGTGAAG; HPRT1 forward, CCTGGCGTCGTGATTAGTGA; and HPRT1 reverse, CGAGCAAGACGTTCAGTCCT. HPRT1 was used as the endogenous reference gene. Relative GDF15 mRNA expression was calculated using the 2^−ΔΔCt^ method, with the mean ΔCt value of the GDF15-L/CAP 0 group used as the calibrator.

### Radiological assessment

Contrast-enhanced abdominal CT was performed at baseline before the initiation of NAC and again after completion of NAC before surgery to evaluate the clinical TNM staging of the tumor. CT scanning was conducted using a triphasic enhancement protocol (arterial, venous, and delayed phases) to ensure a high detection rate of tumors within the gastric wall. CT images were independently analyzed by two experienced radiologists, who measured tumor dimensions (longest/shortest diameters) and lymph node status using multiplanar reconstruction techniques. Lymph nodes greater than 10 mm in short diameter were classified as positive according to the RECIST 1.1 criteria (Eisenhauer et al., 2009).

### Statistical analysis

After the GDF15-L and GDF15-H groups had been defined in the unmatched cohort using the fixed median cutoff described above, propensity score matching (PSM) was performed to reduce potential confounding due to measured baseline differences between the two groups. Propensity scores were estimated using a multivariable logistic regression model incorporating prespecified pre-neoadjuvant clinical characteristics, including sex, age, NRS-2002 score, tumor location, histological differentiation assessed on pretreatment biopsy specimens, Lauren classification, signet-ring cell component, HER2 immunohistochemical score, planned neoadjuvant chemotherapy regimen, and clinical T and N stage. One-to-one nearest-neighbor propensity score matching without replacement was performed using a caliper width of 0.20 standard deviations of the logit of the propensity score. Covariate balance before and after matching was assessed using standardized mean differences (SMDs), with an absolute SMD <0.10 considered indicative of adequate balance. Detailed SMDs before and after matching and the corresponding Love plot are provided in Supplementary Table S1 and Supplementary Figure S2, respectively. Categorical variables are presented as frequencies and percentages, while continuous variables are expressed as medians and IQR. Categorical variables were analyzed using chi-square tests or Fisher’s exact tests, and continuous variables were assessed using t-tests or Mann–Whitney U tests. Kaplan–Meier methods were employed to evaluate overall OS and DFS curves, with log-rank tests used for comparative analysis between different groups. For the focused qRT-PCR validation analysis, relative GDF15 mRNA expression is presented as the mean ± standard deviation. Differences were assessed using two-way ANOVA, with baseline serum GDF15 group (GDF15-L versus GDF15-H) and CAP grade (CAP 0 versus CAP 3) entered as the two factors. Prespecified comparisons between CAP grades 0 and 3 within each serum GDF15 group were performed using Šídák’s multiple-comparisons adjustment. The normality of model residuals and homogeneity of variances were assessed before applying two-way ANOVA. Spearman’s rank correlation analysis was used to evaluate the association between baseline serum GDF15 concentration and tissue GDF15 mRNA expression across the 24 validation samples. All tests were two-sided. Cox regression analysis was conducted to assess the prognostic risk of demographic and clinicopathological features on OS and DFS, incorporating variables with statistically significant differences from univariate analysis into multivariate analysis. When analyzed as a continuous covariate, serum GDF15 was log10-transformed before inclusion in the Cox regression models; accordingly, the corresponding hazard ratio (HR) represents the relative hazard associated with a 10-fold increase in the original serum GDF15 concentration, and HRs are reported with 95% confidence intervals (CIs). The Spearman rank correlation coefficient was calculated to assess the association. A prognostic nomogram was constructed following multivariate Cox regression analysis to visually depict the impact of various factors on patient prognosis. Statistical analyses were performed using R (version 4.4.1, R Foundation for Statistical Computing, Vienna, Austria) and GraphPad Prism 9 (GraphPad Prism Software, Inc., San Diego, California, United States), with a P value of <0.05 considered statistically significant.

## Results

### Baseline characteristics and GDF15 grouping

After PSM, a total of 256 LAGC patients who received NAC were included in the study, and serum levels of GDF15 were analyzed by ELISA. GDF15 levels were significantly elevated in LAGC patients (median: 1735.4 pg/mL, IQR: 1249.5–4908.6) compared with healthy controls (median: 744.14 pg/mL, IQR: 558.9–1033.1; P < 0.001). Patients were stratified into GDF15-L (low expression, n = 128; median: 1247.5 pg/mL, IQR: 1047.9–1517.7) and GDF15-H (high expression, n = 128; median: 4911.6 pg/mL, IQR: 3898.1–5984.4) groups (Figure 1B). Baseline characteristics were summarized in Table 1.

**TABLE 1:** Baseline and clinical characteristics of resectable LAGC patients receiving neoadjuvant chemotherapy after propensity score matching (PSM).

Characteristics	Overall (n = 256)	GDF15-L (n = 128)	GDF15-H (n = 128)	P-value
Gender				0.677
Male	184 (71.9)	90 (70.3)	94 (73.4)	
Female	72 (28.1)	38 (29.7)	34 (26.6)	
Age				***0.045*** *
≥ 60	137 (53.5)	60 (46.9)	77 (60.2)	
< 60	119 (46.5)	68 (53.1)	51 (39.8)	
NRS 2002 Score				0.079
0-2	119 (46.5)	67 (52.3)	52 (40.6)	
≥ 3	137 (53.5)	61 (47.7)	76 (59.4)	
Tumor Location				0.936
Cardia	96 (37.5)	47 (36.7)	49 (38.3)	
Body	44 (17.2)	23 (18.0)	21 (16.4)	
Antrum	116 (45.3)	58 (45.3)	58 (45.3)	
Differentiation Degree				0.040*
Poor	150 (58.6)	65 (50.8)	85 (66.4)	
Moderate	76 (29.7)	45 (35.1)	31 (24.2)	
Well	30 (11.7)	18 (14.1)	12 (9.4)	
Lauran Type				0.931
Intestinal	113 (44.1)	58 (45.3)	55 (43.0)	
Diffuse	94 (36.7)	46 (35.9)	48 (37.5)	
Mixed	49 (19.1)	24 (18.8)	25 (19.5)	
Signet Ring Cell Carcinoma				0.173
Yes	77 (30.1)	33 (25.8)	44 (34.4)	
No	179 (69.9)	95 (74.2)	84 (65.6)	
Her-2				0.559
0	134 (52.3)	67 (52.3)	67 (52.3)	
1	75 (29.3)	36 (28.1)	39 (30.5)	
2	38 (14.8)	22 (17.2)	16 (12.5)	
3	9 (3.5)	3 (2.4)	6 (4.7)	
NAC Regimen				0.395
SOX	195 (76.2)	93 (72.7)	102 (79.7)	
XELOX	48 (18.8)	27 (21.1)	21 (16.4)	
FOLFOX	13 (5.1)	8 (6.2)	5 (3.9)	
No. of NAC cycles				0.81
1	24 (9.3)	12 (9.4)	12 (9.4)	
2	57 (22.3)	26 (20.3)	31 (24.2)	
3	70 (27.3)	34 (26.5)	36 (28.1)	
4	105 (41.0)	56 (43.8)	49 (38.3)	
Clinical T Stage				0.237
T2	50 (19.5)	30 (23.4)	20 (15.6)	
T3	160 (62.5)	78 (61.0)	82 (64.1)	
T4	46 (18.0)	20 (15.6)	26 (20.3)	
Clinical N Status				0.172
N-negative	21 (8.2)	14 (10.9)	7 (5.5)	
N-positive	235 (91.8)	114 (89.1)	121 (94.5)	
Clinical TNM Stage				0.592
II	7 (2.7)	4 (3.2)	3 (2.4)	
III	203 (79.3)	104 (81.2)	99 (77.3)	
IVA	46 (18.0)	20 (15.6)	26 (20.3)	
CEA				0.571
< 5.0 ng/mL	224 (87.5)	110 (85.9)	114 (89.1)	
≥ 5.0 ng/mL	32 (12.5)	18 (14.1)	14 (10.9)	
CA19-9				0.317
< 37 U/mL	228 (89.1)	117 (91.4)	111 (86.7)	
≥ 37 U/mL	28 (10.9)	11 (8.6)	17 (13.3)	
CA72-4				***0.028*** *
< 6.7 μg/L	162 (63.3)	90 (70.3)	72 (56.2)	
≥ 6.7 μg/L	94 (36.7)	38 (29.7)	56 (43.8)	
GDF15 (pg/ml)	1735.4 (1249.5-4908.6)	1247.5 (1047.9-1517.7)	4911.6 (3898.1-5984.4)	***< 0.001*** *

Clinical TNM stage IVA was defined as cT4b, any cN, and cM0; patients with clinical stage IVB disease (any cT, any cN, and cM1) were excluded. All values are expressed as n (%) except for serum GDF15 expression value, which is expressed as the median (IQR: Interquartile range); NAC: neoadjuvant chemotherapy.

* *P < 0.05*.

PSM substantially improved overall covariate balance, with absolute SMDs below 0.10 for most covariates. Residual imbalance remained for age (absolute SMD = 0.140) and histological differentiation (absolute SMD range, 0.154–0.173), as shown in Supplementary Table S1 and Supplementary Figure S2. No significant differences were observed in sex, NRS 2002 score, tumor location, Lauren classification, signet-ring cell features, HER-2 status, NAC regimen/cycles, clinical T/N/TNM stage, or serum CEA/CA19-9 levels between groups (all P > 0.05). However, GDF15-H patients exhibited older age (P = 0.045), poorer tumor differentiation (P = 0.040), and higher serum CA72-4 levels (P = 0.028). In the full, unmatched cohort before PSM, age showed a weak positive correlation with serum GDF15 levels (Spearman’s ρ = 0.137, P = 0.043).

### Survival outcomes and treatment response

With a median follow-up of 53.4 months (range: 3.9–72.6), GDF15-L patients demonstrated superior OS (median: 58.1 vs. 54.0 months; HR = 0.66, 95% CI: 0.45–0.96; P = 0.031) and DFS (median: 47.1 vs. 26.9 months; HR = 0.56, 95% CI: 0.38–0.81; P = 0.002) compared to GDF15-H counterparts (Figure 2). The 3-year OS rates were 70.7% vs. 59.9%, the 5-year OS rates 49.6% vs. 38.8%, and the 3-year DFS rate was 61.3% vs. 43.3%, respectively.

**FIGURE 2:**
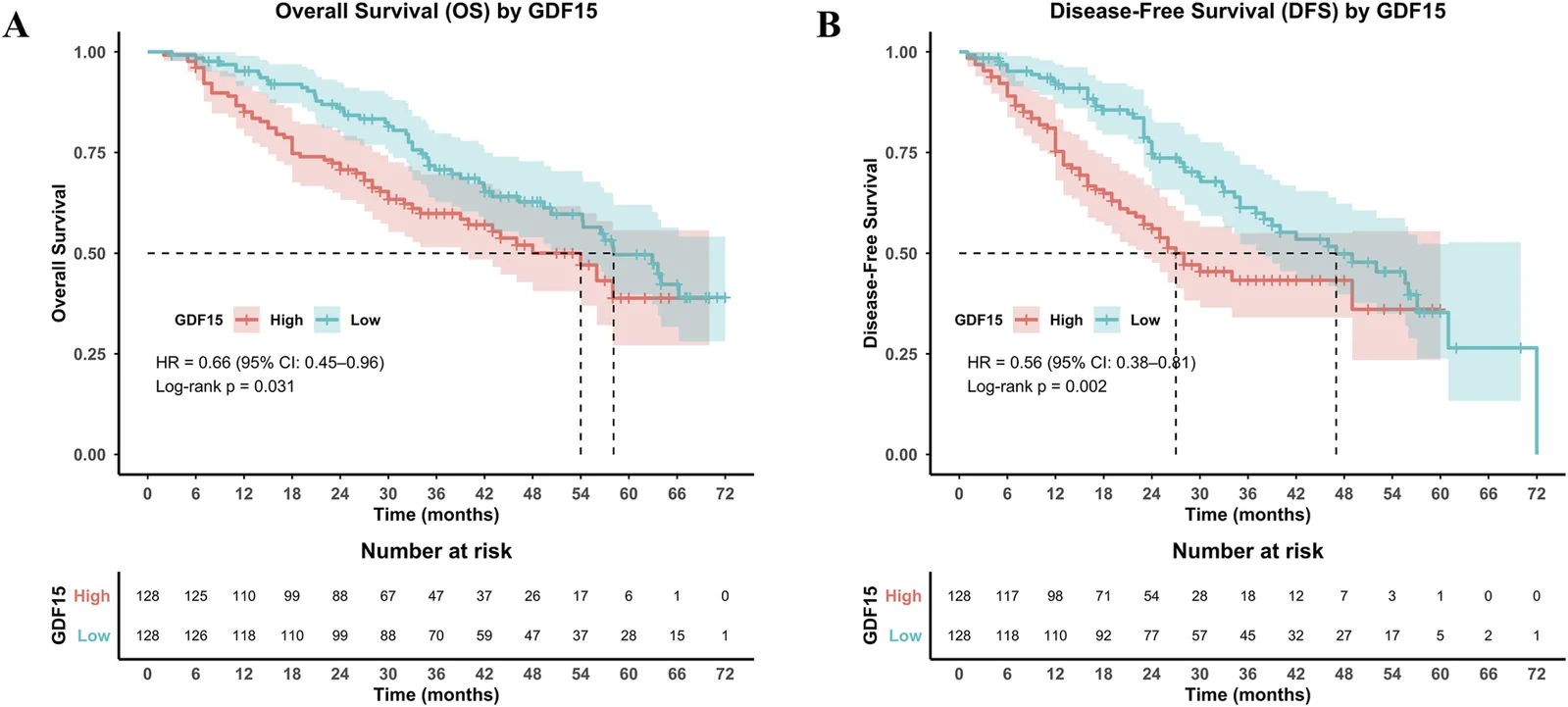
Influence of GDF15 expression on overall survival and disease-free survival of resectable LAGC patients receiving NAC. The Kaplan-Meier curves show overall survival **(A)** and disease-free survival **(B)** of GDF15-H/L patients.

To determine whether baseline serum GDF15 levels reflect intratumoral GDF15 expression, IHC staining was performed on pretreatment endoscopic biopsy specimens obtained before NAC. Intratumoral GDF15 expression was concordant with baseline serum GDF15 levels: stronger GDF15 staining was observed in biopsy specimens from patients in the GDF15-H group than in those from the GDF15-L group (Figure 3). Representative pretreatment biopsy specimens from patients 1 and 2 in the GDF15-H group, who subsequently exhibited CAP grades 2 and 3, and from patients 3 and 4 in the GDF15-L group, who subsequently achieved CAP grades 0 and 1, further illustrated the association between pretreatment GDF15 expression and pathological response to NAC.

**FIGURE 3:**
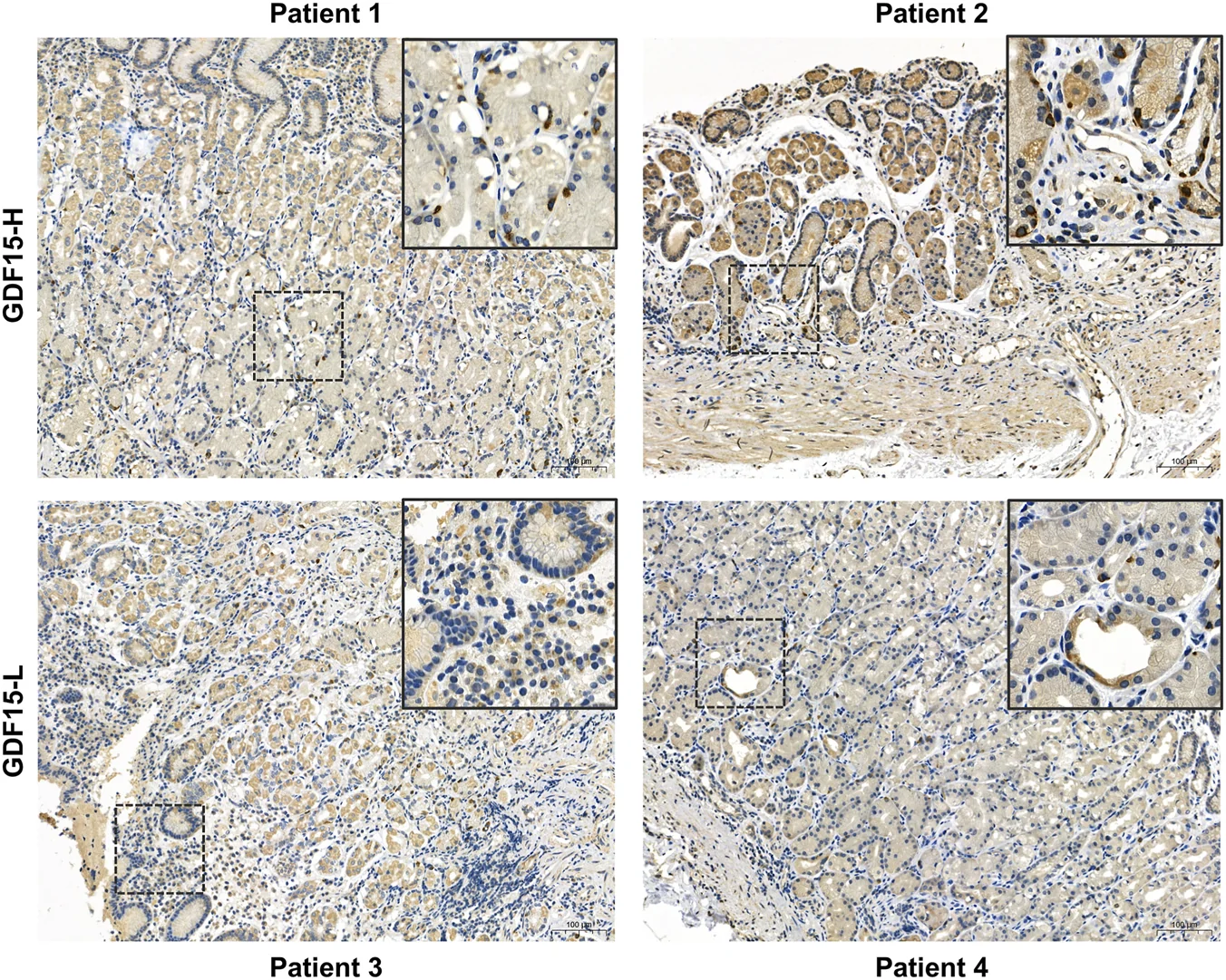
Representative immunohistochemical (IHC) staining of GDF15 in pretreatment endoscopic biopsy specimens obtained before NAC. The IHC staining results illustrate the expression status of GDF15 in the tumor interior of patients in GDF15-H/L groups. The dotted lines show the magnified area. Scale bar, 100 μm.

These qualitative IHC observations were further evaluated quantitatively by qRT-PCR in a focused subset comprising the most contrasting pathological response phenotypes. In the focused qRT-PCR validation subset, the relative tissue GDF15 mRNA expression levels were 0.92 ± 0.31 in the GDF15-L/CAP 0 subgroup, 1.76 ± 0.30 in the GDF15-L/CAP 3 subgroup, 2.32 ± 0.70 in the GDF15-H/CAP 0 subgroup, and 3.40 ± 0.84 in the GDF15-H/CAP 3 subgroup. Two-way ANOVA demonstrated significant main effects of baseline serum GDF15 group (F [1,20] = 40.14, P < 0.001) and CAP grade (F [1,20] = 15.94, P < 0.001). Šídák-adjusted comparisons showed significantly higher tissue GDF15 mRNA expression in CAP grade 3 than in CAP grade 0 within both the GDF15-L group (P = 0.045) and the GDF15-H group (P = 0.009). Across all 24 validation samples, baseline serum GDF15 concentration was strongly positively correlated with tissue GDF15 mRNA expression (Spearman’s ρ = 0.90, P < 0.001; Supplementary Figure S1). These findings provide quantitative tissue-level support for the concordance between circulating and intratumoral GDF15 expression and its association with pathological response to NAC. And this may help identify patients at increased risk of poor pathological response and unfavorable long-term survival outcomes.

Representative CT images (Figure 4) from matched patients who underwent 4 cycles of the SOX regimen revealed marked tumor regression in the GDF15-L patients, while the treatment response was not significant in the GDF15-H patients. No statistically significant differences in perineural or vascular invasion were observed between the groups. Although all patients were classified as cM0 before NAC, distant metastases that were absent at baseline were newly detected during or after NAC in 1 of 128 patients in the GDF15-L group and 9 of 128 patients in the GDF15-H group (P = 0.024). Significant downstaging in ypT (P = 0.006), ypN (P = 0.018), and ypTNM (P = 0.011) stages was observed in GDF15-L patients.

**FIGURE 4:**
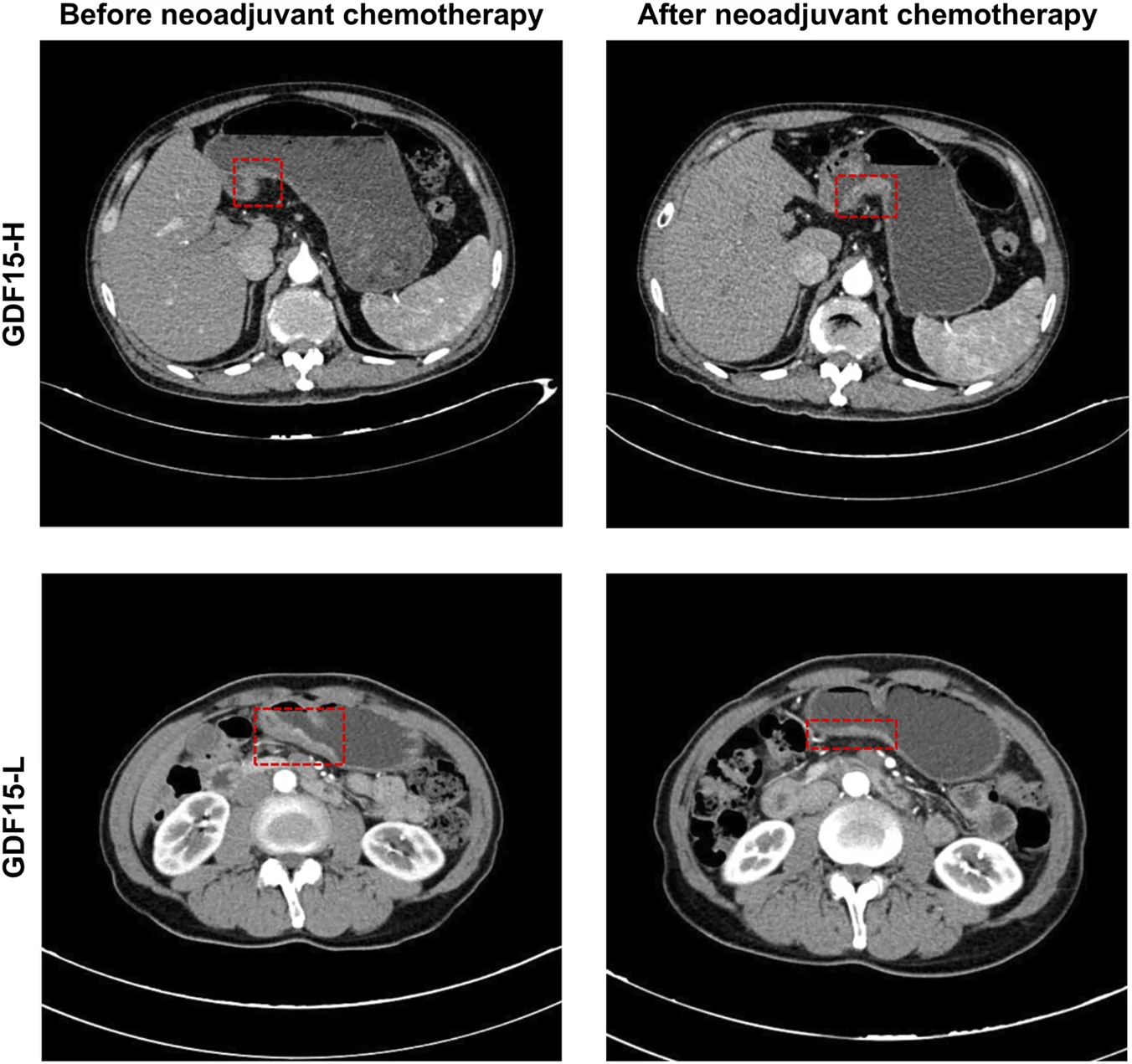
Representative abdominal enhanced CT images of patients. From left to right, the images of each row show the change of tumor size in GDF15-H/L patients before and after receiving NAC. The tumor region is marked by red dashed boxes.

In the resected specimens, the absence of residual viable cancer cells after neoadjuvant chemotherapy was classified as CAP degree 0, indicating a pathological complete response (pCR). In contrast, extensive residual cancer with no evident tumor regression was classified as CAP degree 3, suggesting minimal or no tumor regression after NAC and indicating a poor treatment response. The proportions of CAP degree 0, 1, 2, and 3 in the GDF15-L and GDF15-H groups were 21.1% vs. 7.8%, 24.2% vs. 9.4%, 25.8% vs. 41.6%, and 28.9% vs. 42.2%, respectively. Collectively, these findings indicate that low baseline serum GDF15 levels were associated with a more favorable pathological response to NAC (Table 2).

**TABLE 2:** Patient’s treatment response after neoadjuvant chemotherapy.

Characteristics	GDF15-L (n = 128)	GDF15-H (n = 128)	P-value
Neurological invasion			0.106
No	103 (80.5)	92 (71.9)	
Yes	25 (19.5)	36 (28.1)	
Vascular invasion			0.248
No	102 (79.7)	85 (66.4)	
Yes	26 (20.3)	43 (33.6)	
ypT stage			***0.006*** *
T0	27 (21.1)	10 (7.8)	
T1	30 (23.4)	18 (14.1)	
T2	33 (25.8)	31 (24.2)	
T3	28 (21.9)	46 (35.9)	
T4	10 (7.8)	23 (18.0)	
ypN stage			***0.018*** *
N0	27 (21.1)	23 (18.0)	
N1	83 (64.8)	66 (51.6)	
N2	13 (10.2)	29 (22.6)	
N3	5 (3.9)	10 (7.8)	
Newly detected distant metastasis during or after NAC			***0.024*** *
No	127 (99.2)	119 (93.0)	
Yes	1 (0.8)	9 (7.0)	
ypTNM stage			***0.011*** *
0	7 (5.5)	5 (3.9)	
I	14 (10.9)	12 (9.4)	
II	46 (35.9)	25 (19.5)	
III	54 (42.2)	68 (53.1)	
IV	7 (5.5)	18 (14.1)	
CAP degree			***< 0.001*** *
0	27 (21.1)	10 (7.8)	
1	31 (24.2)	12 (9.4)	
2	33 (25.8)	52 (41.6)	
3	37 (28.9)	54 (42.2)	

All patients were cM0 at baseline. “Yes” indicates distant metastasis absent at baseline but first detected between NAC initiation and surgery by post-NAC imaging, intraoperative findings, and/or histopathological examination. yp: postoperative pathological stage after neoadjuvant chemotherapy; CAP: the tumor regression grading (TRG) system proposed by the College of American Pathologists.

* *P < 0.05*.

### Survival analysis

Univariate Cox regression identified GDF15 group, GDF15 expression level, age, vascular invasion, ypT/N/TNM stage, distant metastasis, and CAP degree as prognostic factors for OS and DFS (Table 3). Multivariate analysis revealed that a higher log10-transformed serum GDF15 concentration (HR = 2.09, 95% CI: 1.12–3.92, P = 0.021) and CAP degree (HR = 1.71, 95% CI: 1.08–2.7, P = 0.022) were independent risk factors for OS, while younger age (HR = 0.61, 95% CI: 0.41–0.91, P = 0.016) was identified as a protective factor. In addition, high GDF15 expression (HR = 1.95, 95% CI: 1.02–3.51, P = 0.048), younger age (HR = 0.64, 95% CI: 0.43–0.95, P = 0.027), vascular invasion (HR = 1.59, 95% CI: 1.05–2.41, P = 0.029), and elevated CAP degree (HR = 2.02, 95% CI: 1.27–3.2, P = 0.003) were independently associated with DFS (Table 4).

**TABLE 3:** Univariable Cox regression analysis of factors associated with survival of patients.

Variables	OS		DFS	
	HR (95%CI)	P-value	HR (95%CI)	P-value
GDF15 group (GDF15-H vs*.* GDF15-L)	1.57 (1.03–2.32)	***0.028*** *	1.63 (1.18–2.97)	***0.022*** *
Serum GDF15 concentration, log10-transformed (per 1-unit increase)	2.79 (1.5–5.17)	***0.001*** *	2.96 (1.57–5.59)	***0.001*** *
Age (<60 vs*.* ≥ 60)	0.64 (0.44–0.94)	***0.024*** *	0.68 (0.46–1)	***0.047*** *
Tumor location (cardia vs*.* body/Antrum)	1.06 (0.72–1.55)	0.769	1.01 (0.69–1.47)	0.978
Differentiation degree (poor vs*.* moderate/Well)	0.76 (0.52–1.11)	0.156	0.81 (0.55–1.18)	0.274
Lauran type (intestinal vs*.* diffuse/Mixed)	0.73 (0.5–1.06)	0.095	0.75 (0.52–1.1)	0.139
Signet ring cell carcinoma (yes vs*.* no)	1.02 (0.68–1.54)	0.911	1.07 (0.71–1.61)	0.737
Her-2 (0/1 vs*.* 2/3)	0.91 (0.57–1.46)	0.696	0.92 (0.57–1.48)	0.72
NAC regimen (SOX vs*.* XELOX/FOLFOX)	1.39 (0.94–2.03)	0.095	1.36 (0.94–1.97)	0.102
No. of NAC cycles (1/2 vs*.* 3/4)	1.15 (0.76–1.74)	0.505	1.1 (0.73–1.66)	0.651
Clinical T stage (T3/4 vs*.* T2)	1.51 (0.91–2.51)	0.111	1.63 (0.96–2.71)	0.058
Clinical N Status (N-positive vs*.* N-negative)	1.22 (0.62–2.4)	0.574	1.2 (0.56–2.56)	0.634
Clinical TNM stage (III/IV vs*.* II)	3.72 (0.6–22.97)	0.158	3.75 (0.5–28.06)	0.198
CEA (<5.0 ng/mL vs*.* ≥ 5.0 ng/mL)	0.81 (0.48–1.37)	0.438	0.86 (0.5–1.47)	0.583
CA19-9 (<37 U/mL vs*.* ≥ 37 U/mL)	0.89 (0.46–1.71)	0.72	0.97 (0.5–1.89)	0.934
CA72-4 (<6.7 μg/L vs*.* ≥ 6.7 μg/L)	0.79 (0.54–1.15)	0.216	0.77 (0.53–1.13)	0.179
Neurological invasion (yes vs*.* no)	1.21 (0.78–1.87)	0.386	1.38 (0.89–2.13)	0.151
Vascular invasion (yes vs*.* no)	1.49 (1–2.21)	***0.048*** *	1.88 (1.27–2.78)	***0.002*** *
ypT stage (T3/4 vs*.* T0/1/2)	1.54 (1.05–2.25)	***0.027*** *	1.67 (1.14–2.45)	***0.008*** *
ypN stage (N2/3 vs*.* N0/1)	1.56 (1.03–2.37)	***0.038*** *	1.74 (1.14–2.65)	***0.011*** *
Newly detected distant metastasis during or after NAC (yes vs*.* no)	5.5 (2.07–14.59)	***0.001*** *	9.09 (4.27–19.37)	***< 0.001*** *
ypTNM stage (III/IV vs*.* 0/i/II)	1.58 (1.08–2.32)	***0.018*** *	1.79 (1.23–2.62)	***0.003*** *
CAP degree (2/3 vs*.* 0/1)	1.83 (1.18–2.84)	***0.007*** *	2.23 (1.44–3.46)	***< 0.001*** *

NAC: neoadjuvant chemotherapy; yp: postoperative pathological stage after neoadjuvant chemotherapy; CAP: the tumor regression grading (TRG) system proposed by the College of American Pathologists; HR: hazard ratio; CI: confidence interval.

* *P < 0.05*.

**TABLE 4:** Multivariable Cox regression analysis of factors associated with survival of patients.

Variables	OS		DFS	
	HR (95%CI)	P-value	HR (95%CI)	P-value
Serum GDF15 concentration, log10-transformed (per 1-unit increase)	2.09 (1.12–3.92)	***0.021*** *	1.95 (1.02–3.51)	***0.048*** *
Age (<60 vs*.* ≥ 60)	0.61 (0.41–0.91)	***0.016*** *	0.64 (0.43–0.95)	***0.027*** *
Vascular invasion (yes vs*.* no)	1.33 (0.88–2.01)	0.174	1.59 (1.05–2.41)	***0.029*** *
CAP degree (2/3 vs*.* 0/1)	1.71 (1.08–2.7)	***0.022*** *	2.02 (1.27–3.2)	***0.003*** *

CAP: the tumor regression grading (TRG) system proposed by the College of American Pathologists; HR: hazard ratio; CI: confidence interval.

* *P < 0.05*.

### Construction of prognostic nomogram associated with GDF15

Incorporating the aforementioned prognostic factors for OS and DFS, the constructed prognostic nomogram visually illustrates the impact of each variable on patient prognosis, facilitating the assessment of 1-year, 3-year, and 5-year OS and DFS for patients with LAGC undergoing NAC (Figure 5). The nomogram assigns scores to each prognostic variable based on regression coefficients, the results revealing that GDF15 expression is the primary factor influencing OS and DFS, followed by CAP degree, with age having a moderate effect on OS and DFS. Each prognostic factor is assigned a score on a point scale; due to the non-normal distribution and variability of serum GDF15 expression values, a logarithmic transformation (log10) was applied to stabilize the model. By summing the total scores on the total point scale, the corresponding 1-year, 3-year, and 5-year survival rates can be estimated.

**FIGURE 5:**
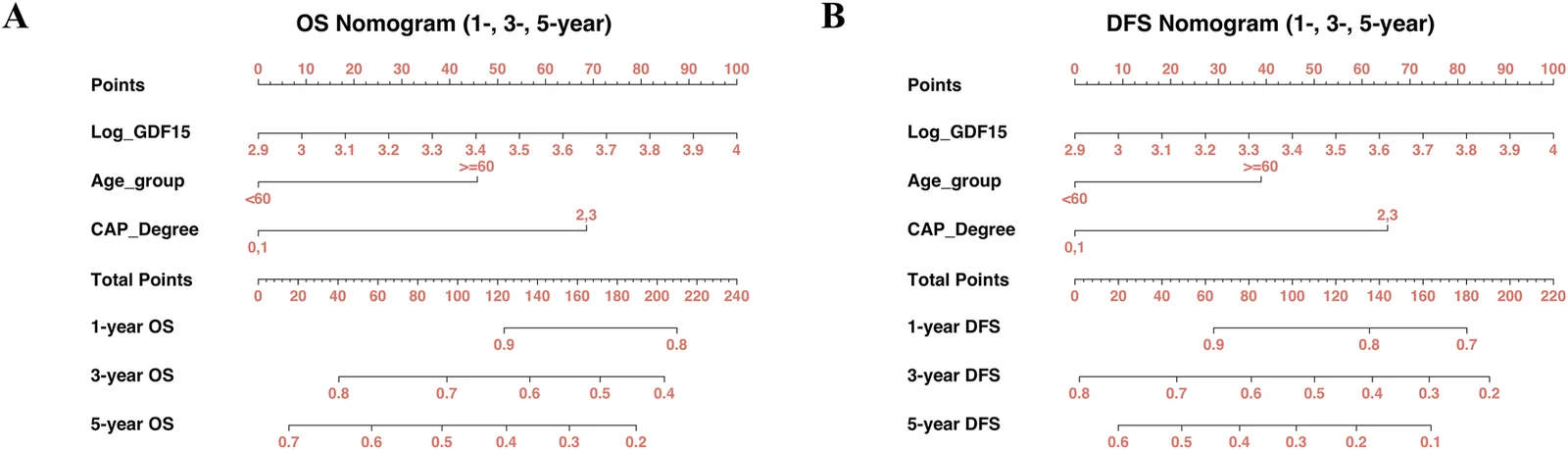
Prognostic nomograms to predict the OS and DFS of resectable LAGC patients receiving NAC. **(A)** Prognostic nomograms to predict the overall survival (OS). **(B)** Prognostic nomograms to predict the disease-free survival (DFS). CAP: the tumor regression grading (TRG) system proposed by the College of American Pathologists.

## Discussion

Patients with resectable LAGC frequently encounter dilemmas, including substantial tumor burden and suboptimal outcomes with postoperative adjuvant therapy alone. Although multiple phase III clinical trials have validated the survival benefits of NAC in LAGC patients (Cunningham et al., 2006; Al-Batran et al., 2019; Kang et al., 2021; Kang et al., 2024; Wang X. et al., 2021), the persistent heterogeneity in therapeutic efficacy across individuals results in tumor progression with missed surgical opportunities, early recurrence, or unnecessary treatment toxicity for a considerable proportion of non-responders (Yin et al., 2024). This underscores the imperative need for novel biomarkers to optimize therapeutic decision-making. Liquid biopsy has emerged as a promising prognostic monitoring approach, enabling minimally invasive detection of circulating biomarkers in blood or urine with advantages of high sensitivity, specificity, and reproducibility. This methodology encompasses various biomarkers including circulating tumor cells (CTCs), circulating tumor DNA (ctDNA), and exosomes, among which serum biomarkers constitute a critical component (Wu et al., 2020). However, conventional biomarkers such as CA72-4, despite their partial utility in GC screening, demonstrate insufficient sensitivity and specificity for clinical demands. Furthermore, while ctDNA shows superiority in early diagnosis and dynamic monitoring, its widespread clinical application remains constrained by technical complexity and elevated costs (Cao et al., 2022).

To our knowledge, this represents the first investigation establishing the association between serum GDF15 levels and NAC therapeutic response/survival outcomes in LAGC patients. As previously mentioned, GDF15 has been identified as overexpressed in multiple malignancies, closely correlating with tumor invasion, metastasis, and chemoresistance. Potential mechanisms may involve mitochondrial dysfunction triggering subsequent cellular senescence and the development of senescence-associated secretory phenotype (SASP), or HIF1α-mediated recruitment of M2 tumor-associated macrophages (TAMs) within the tumor microenvironment, thereby conferring chemoprotection (Wang et al., 2024; Yu et al., 2020). In our cohort, elevated GDF15 expression correlated with advanced age and poorer tumor differentiation. We postulate that this association may stem from age-related cellular senescence, which is no longer considered merely a tumor-suppressive mechanism but rather a promoter of adverse biological behaviors (Demaria et al., 2017). Chemotherapy has been recognized as a potent mechanism for inducing cellular senescence (Saleh et al., 2020), with GDF15 established as a core senescence-associated biomarker (St Sauver et al., 2023). Notably, the qualitative IHC observations were complemented by qRT-PCR analysis in a focused subset of patients with the most contrasting pathological response phenotypes. Tissue GDF15 mRNA expression was higher in patients with CAP grade 3 than in those with CAP grade 0 within both serum GDF15 groups, and tissue GDF15 mRNA expression was strongly correlated with baseline serum GDF15 concentration. These results provide orthogonal molecular support for the biological concordance between circulating and intratumoral GDF15 expression and strengthen the rationale for evaluating serum GDF15 as a response-associated biomarker. Nevertheless, this focused validation does not independently establish clinical diagnostic performance or differential benefit from NAC.

Prior studies have implicated GDF15 in prognosticating various malignancies, yet its specific role in NAC-treated LAGC patients remains underexplored. Our findings demonstrate that LAGC patients with elevated serum GDF15 exhibit inferior OS and DFS following NAC, aligning with observations by Xu et al. in non-small cell lung cancer (NSCLC) (Xu et al., 2021) and Zhao et al. in epithelial ovarian cancer (EOC) (Zhao et al., 2018), that GDF15 overexpression was significantly correlated with chemoresistance and reduced survival. Besides, although there was a weak correlation between age and GDF15 expression, our multivariate analysis confirmed their independent prognostic associations. This conclusion resonates with Teramoto et al.'s findings (Teramoto et al., 2024), where GDF15 independently predicted cardiovascular mortality and heart failure despite age-associated elevation, but the mechanistic complexities warranting further experimental investigation.

Beyond prognostic implications, the potential value of GDF15 for treatment-response risk stratification holds greater clinical significance, as shorter survival periods are generally associated with suboptimal treatment outcomes. Our radiologic and pathologic results revealed enhanced tumor regression on CT evaluation, lower postoperative CAP degree, and higher pCR rates in the GDF15-L cohort, supporting the potential of GDF15 to identify patients at risk of poor response. In terms of its mechanism, a study on colorectal cancer shown that inhibiting GDF15 might enhance the efficacy of 5-fluorouracil (5-FU) chemotherapy by regulating ferroptosis pathways, with GDF15 inhibition combined with 5-FU resulting in smaller tumor volumes (Huang et al., 2024). In hepatocellular carcinoma, GDF15 may compromise anti-tumor immunity through Treg cell activation via CD48 receptor interaction (Wang Z. et al., 2021). These studies indirectly support the findings of our research, however, the effects of microenvironmental differences due to tissue specificity still need to be taken into account. Additionally, Zheng et al. (2020) discovered that GDF15 induces resistance by promoting angiogenesis, and the use of the Smad signaling pathway activator TGF-β1 can partially mitigate these effects, potentially explaining the higher incidence of newly detected distant metastasis during or after NAC in GDF15-H patients, particularly for DFS, where vascular invasion is an additional independent risk factor.

Current clinical practice in NAC faces a “one-size-fits-all” dilemma. Our findings suggest using serum GDF15 levels for risk stratification. Low-GDF15 patients may derive maximal benefit from standard NAC, whereas high-GDF15 individuals might benefit from early surgical intervention or intensified regimens incorporating immunotherapy and targeted agents. Notably, our recommendation does not categorically exclude NAC for high-GDF15 patients, but rather advocates for treatment intensification based on their biomarker profile. While these patients may exhibit suboptimal responses to conventional NAC alone, strategic combinations with checkpoint inhibitors, angiogenesis blockers, or emerging GDF15-targeted therapies could potentially overcome resistance mechanisms. However, prospective validation remains essential prior to clinical implementation. Encouragingly, recent phase I/IIa trials of visugromab (CTL-002), a GDF15-neutralizing antibody, demonstrated durable responses in PD-1/PD-L1-resistant NSCLC and urothelial carcinoma (Melero et al., 2025). Other anti-GDF15 monoclonal antibodies (e.g., ponsegromab, AZD8853) have also shown favorable safety profiles in solid tumors and cancer cachexia clinical trials (Crawford et al., 2024; Carneiro et al., 2025). This evolving therapeutic landscape suggests that high-GDF15 patients may particularly benefit from clinical trials evaluating NAC-anti-GDF15 combination strategies, potentially preserving chemotherapy sensitivity while counteracting GDF15-mediated immunosuppression.

We acknowledge that this study has several limitations. The retrospective single-center design and moderate sample size may cause potential selection bias and higher risk ratios. Ethnic and geographic homogeneity and predominant SOX regimen usage may limit generalizability, necessitating multicenter prospective validation. Because all patients received NAC and no alternative-treatment comparator group was included, the present study cannot directly determine whether GDF15 predicts differential benefit from NAC. Furthermore, real-time monitoring of GDF15 dynamics during NAC was not performed. In addition, the qRT-PCR validation was conducted in a relatively small, focused subset selected according to the availability of frozen pretreatment biopsy tissue and the two extreme CAP response categories. Consequently, the observed serum–tissue correlation may be influenced by the selected phenotype spectrum and should be regarded as exploratory. Prospective validation in a larger, consecutively enrolled and unselected cohort is required. Despite these constraints, our findings substantiate serum GDF15’s potential value for treatment-response and prognostic stratification in LAGC patients, providing a new insight for personalized neoadjuvant strategies.

## Conclusion

In conclusion, baseline serum GDF15 was consistently associated with radiological response, pathological tumor regression, and survival outcomes in patients with resectable LAGC receiving NAC. As a minimally invasive and readily measurable marker, GDF15 shows promise for pretreatment response-risk and prognostic stratification and may help identify patients requiring closer response assessment and timely multidisciplinary reassessment. However, because all patients received NAC, this study cannot definitively determine whether GDF15 predicts differential benefit from NAC. Prospective biomarker-stratified studies are needed to validate its clinical utility and evaluate alternative or intensified treatment strategies for high-risk patients.

## Data Availability

The original contributions presented in the study are included in the article/Supplementary Material, further inquiries can be directed to the corresponding authors.

## Supplementary material

SUPPLEMENTARY FIGURE S1 Quantitative real-time PCR validation of GDF15 mRNA expression in pretreatment endoscopic biopsy specimens. **(A)** Relative GDF15 mRNA expression in pretreatment frozen endoscopic biopsy specimens from the GDF15-L/CAP 0, GDF15-L/CAP 3, GDF15-H/CAP 0, and GDF15-H/CAP 3 subgroups (n = 6 per subgroup). GDF15 expression was normalized to HPRT1 and calculated using the 2^−ΔΔCt^ method. Bars represent the mean ± standard deviation, and each dot represents one patient. Differences were evaluated using two-way ANOVA with serum GDF15 group and CAP grade as the two factors, followed by Šídák-adjusted comparisons between CAP grades 0 and 3 within each serum GDF15 group. The uppermost bracket indicates the main effect of baseline serum GDF15 group. **(B)** Spearman correlation between baseline serum GDF15 concentration and relative tissue GDF15 mRNA expression across all 24 validation samples. The dashed line represents a least-squares linear fit for visualization only and was not used to calculate Spearman’s correlation coefficient. Spearman’s ρ = 0.90, P < 0.001. CAP grade 0 indicates the absence of residual viable tumor cells after NAC, whereas CAP grade 3 indicates minimal or no pathological tumor response. * P < 0.05, ** P < 0.01, and *** P < 0.001.

SUPPLEMENTARY FIGURE S2 Love plot showing covariate balance before and after propensity score matching. Red circles and blue triangles represent the absolute standardized mean differences before and after matching, respectively. The vertical dashed line indicates the prespecified balance threshold of 0.10.
