# Supplementary material for: Serum GDF15 as a potential biomarker for treatment response and survival in patients with resectable locally advanced gastric cancer undergoing neoadjuvant chemotherapy
Source: Front Mol Biosci. 2026 Oct 7;13:1948454. doi: 10.3389/fmolb.2026.1948454 (PMC13683077; doi:10.3389/fmolb.2026.1948454)
Supplement: Supplementary file 1 [file Table1.docx]

Table 1. Baseline and clinical characteristics of resectable LAGC patients receiving neoadjuvant chemotherapy after propensity score matching (PSM)

| **Characteristics** | **N**  **n = 256** | **GDF15-L**  **n = 128** | **GDF15-H**  **n = 128** | **P-value** |
| --- | --- | --- | --- | --- |
| Gender |  |  |  | 0.677 |
| Male | 184 (71.9) | 90 (70.3) | 94 (73.4) |  |
| Female | 72 (28.1) | 38 (29.7) | 34 (26.6) |  |
| Age |  |  |  | ***0.045**** |
| ≥ 60 | 137 (53.5) | 60 (46.9) | 77 (60.2) |  |
| < 60 | 119 (46.5) | 68 (53.1) | 51 (39.8) |  |
| NRS 2002 Score |  |  |  | 0.079 |
| 0-2 | 119 (46.5) | 67 (52.3) | 52 (40.6) |  |
| ≥ 3 | 137 (53.5) | 61 (47.7) | 76 (59.4) |  |
| Tumor Location |  |  |  | 0.936 |
| Cardia | 96 (37.5) | 47 (36.7) | 49 (38.3) |  |
| Body | 44 (17.2) | 23 (18.0) | 21 (16.4) |  |
| Antrum | 116 (45.3) | 58 (45.3) | 58 (45.3) |  |
| Differentiation Degree |  |  |  | ***0.040**** |
| Poor | 150 (58.6) | 65 (50.8) | 85 (66.4) |  |
| Moderate | 76 (29.7) | 45 (35.1) | 31 (24.2) |  |
| Well | 30 (11.7) | 18 (14.1) | 12 (9.4) |  |
| Lauran Type |  |  |  | 0.931 |
| Intestinal | 113 (44.1) | 58 (45.3) | 55 (43.0) |  |
| Diffuse | 94 (36.7) | 46 (35.9) | 48 (37.5) |  |
| Mixed | 49 (19.1) | 24 (18.8) | 25 (19.5) |  |
| Signet Ring Cell Carcinoma |  |  |  | 0.173 |
| Yes | 77 (30.1) | 33 (25.8) | 44 (34.4) |  |
| No | 179 (69.9) | 95 (74.2) | 84 (65.6) |  |
| Her-2 |  |  |  | 0.559 |
| 0 | 134 (52.3) | 67 (52.3) | 67 (52.3) |  |
| 1 | 75 (29.3) | 36 (28.1) | 39 (30.5) |  |
| 2 | 38 (14.8) | 22 (17.2) | 16 (12.5) |  |
| 3 | 9 (3.5) | 3 (2.4) | 6 (4.7) |  |
| NAC Regimen |  |  |  | 0.395 |
| SOX | 195 (76.2) | 93 (72.7) | 102 (79.7) |  |
| XELOX | 48 (18.8) | 27 (21.1) | 21 (16.4) |  |
| FOLFOX | 13 (5.1) | 8 (6.2) | 5 (3.9) |  |
| No. of NAC cycles |  |  |  | 0.81 |
| 1 | 24 (9.3) | 12 (9.4) | 12 (9.4) |  |
| 2 | 57 (22.3) | 26 (20.3) | 31 (24.2) |  |
| 3 | 70 (27.3) | 34 (26.5) | 36 (28.1) |  |
| 4 | 105 (41.0) | 56 (43.8) | 49 (38.3) |  |
| Clinical T Stage |  |  |  | 0.237 |
| T2 | 50 (19.5) | 30 (23.4) | 20 (15.6) |  |
| T3 | 160 (62.5) | 78 (61.0) | 82 (64.1) |  |
| T4 | 46 (18.0) | 20 (15.6) | 26 (20.3) |  |
| Clinical N Status |  |  |  | 0.172 |
| N-negative | 21 (8.2) | 14 (10.9) | 7 (5.5) |  |
| N-positive | 235 (91.8) | 114 (89.1) | 121 (94.5) |  |
| Clinical TNM Stage |  |  |  | 0.592 |
| II | 7 (2.7) | 4 (3.2) | 3 (2.4) |  |
| III | 203 (79.3) | 104 (81.2) | 99 (77.3) |  |
| IVA | 46 (18.0) | 20 (15.6) | 26 (20.3) |  |
| CEA |  |  |  | 0.571 |
| < 5.0 ng/mL | 224 (87.5) | 110 (85.9) | 114 (89.1) |  |
| ≥ 5.0 ng/mL | 32 (12.5) | 18 (14.1) | 14 (10.9) |  |
| CA19-9 |  |  |  | 0.317 |
| < 37 U/mL | 228 (89.1) | 117 (91.4) | 111 (86.7) |  |
| ≥ 37 U/mL | 28 (10.9) | 11 (8.6) | 17 (13.3) |  |
| CA72-4 |  |  |  | ***0.028**** |
| < 6.7 μg/L | 162 (63.3) | 90 (70.3) | 72 (56.2) |  |
| ≥ 6.7 μg/L | 94 (36.7) | 38 (29.7) | 56 (43.8) |  |
| GDF15 (pg/ml) | 1735.4  (1249.5-4908.6) | 1247.5  (1047.9-1517.7) | 4911.6  (3898.1-5984.4) | ***< 0.001**** |

Clinical TNM stage IVA was defined as cT4b, any cN, and cM0; patients with clinical stage IVB disease (any cT, any cN, and cM1) were excluded. All values are expressed as n (%) except for serum GDF15 expression value, which is expressed as the median (IQR: Interquartile range); NAC: neoadjuvant chemotherapy; ** P < 0.05*
