# Supplementary material for: Serum GDF15 as a potential biomarker for treatment response and survival in patients with resectable locally advanced gastric cancer undergoing neoadjuvant chemotherapy
Source: Front Mol Biosci. 2026 Oct 7;13:1948454. doi: 10.3389/fmolb.2026.1948454 (PMC13683077; doi:10.3389/fmolb.2026.1948454)
Supplement: Supplementary file 2 [file Table2.DOCX]

Table 2. Patient's treatment response after neoadjuvant chemotherapy

| **Characteristics** | **GDF15-L (n = 128)** | **GDF15-H (n = 128)** | **P-value** |
| --- | --- | --- | --- |
| Neurological Invasion |  |  | 0.106 |
| No | 103 (80.5) | 92 (71.9) |  |
| Yes | 25 (19.5) | 36 (28.1) |  |
| Vascular Invasion |  |  | 0.248 |
| No | 102 (79.7) | 85 (66.4) |  |
| Yes | 26 (20.3) | 43 (33.6) |  |
| ypT Stage |  |  | ***0.006**** |
| T0 | 27 (21.1) | 10 (7.8) |  |
| T1 | 30 (23.4) | 18 (14.1) |  |
| T2 | 33 (25.8) | 31 (24.2) |  |
| T3 | 28 (21.9) | 46 (35.9) |  |
| T4 | 10 (7.8) | 23 (18.0) |  |
| ypN Stage |  |  | ***0.018**** |
| N0 | 27 (21.1) | 23 (18.0) |  |
| N1 | 83 (64.8) | 66 (51.6) |  |
| N2 | 13 (10.2) | 29 (22.6) |  |
| N3 | 5 (3.9) | 10 (7.8) |  |
| Newly detected distant metastasis during or after NAC |  |  | ***0.024**** |
| No | 127 (99.2) | 119 (93.0) |  |
| Yes | 1 (0.8) | 9 (7.0) |  |
| ypTNM Stage |  |  | ***0.011**** |
| 0 | 7 (5.5) | 5 (3.9) |  |
| I | 14 (10.9) | 12 (9.4) |  |
| II | 46 (35.9) | 25 (19.5) |  |
| III | 54 (42.2) | 68 (53.1) |  |
| IV | 7 (5.5) | 18 (14.1) |  |
| CAP Degree |  |  | ***< 0.001**** |
| 0 | 27 (21.1) | 10 (7.8) |  |
| 1 | 31 (24.2) | 12 (9.4) |  |
| 2 | 33 (25.8) | 52 (41.6) |  |
| 3 | 37 (28.9) | 54 (42.2) |  |

All patients were cM0 at baseline. “Yes” indicates distant metastasis absent at baseline but first detected between NAC initiation and surgery by post-NAC imaging, intraoperative findings, and/or histopathological examination. yp: postoperative pathological stage after neoadjuvant chemotherapy; CAP: the tumor regression grading (TRG) system proposed by the College of American Pathologists

** P < 0.05*
