# Supplementary material for: Serum GDF15 as a potential biomarker for treatment response and survival in patients with resectable locally advanced gastric cancer undergoing neoadjuvant chemotherapy
Source: Front Mol Biosci. 2026 Oct 7;13:1948454. doi: 10.3389/fmolb.2026.1948454 (PMC13683077; doi:10.3389/fmolb.2026.1948454)
Supplement: Supplementary file 3 [file Table3.DOCX]

Table 3. Univariable Cox regression analysis of factors associated with survival of patients

| **Variables** | **OS** | |  | **DFS** | |
| --- | --- | --- | --- | --- | --- |
|  | **HR (95%CI)** | **P-value** |  | **HR (95%CI)** | **P-value** |
| GDF15 Group (GDF15-H *vs* GDF15-L) | 1.57 (1.03-2.32) | ***0.028**** |  | 1.63 (1.18-2.97) | ***0.022**** |
| Serum GDF15 concentration, log10-transformed (per 1-unit increase) | 2.79 (1.5-5.17) | ***0.001**** |  | 2.96 (1.57-5.59) | ***0.001**** |
| Age (< 60 *vs* ≥ 60) | 0.64 (0.44-0.94) | ***0.024**** |  | 0.68 (0.46-1) | ***0.047**** |
| Tumor Location (Cardia *vs* Body/Antrum) | 1.06 (0.72-1.55) | 0.769 |  | 1.01 (0.69-1.47) | 0.978 |
| Differentiation Degree (Poor *vs* Moderate/Well) | 0.76 (0.52-1.11) | 0.156 |  | 0.81 (0.55-1.18) | 0.274 |
| Lauran Type (Intestinal *vs* Diffuse/Mixed) | 0.73 (0.5-1.06) | 0.095 |  | 0.75 (0.52-1.1) | 0.139 |
| Signet Ring Cell Carcinoma (Yes *vs* No) | 1.02 (0.68-1.54) | 0.911 |  | 1.07 (0.71-1.61) | 0.737 |
| Her-2 (0/1 *vs* 2/3) | 0.91 (0.57-1.46) | 0.696 |  | 0.92 (0.57-1.48) | 0.72 |
| NAC Regimen (SOX *vs* XELOX/FOLFOX) | 1.39 (0.94-2.03) | 0.095 |  | 1.36 (0.94-1.97) | 0.102 |
| No. of NAC cycles (1/2 *vs* 3/4) | 1.15 (0.76-1.74) | 0.505 |  | 1.1 (0.73-1.66) | 0.651 |
| Clinical T Stage (T3/4 *vs* T2) | 1.51 (0.91-2.51) | 0.111 |  | 1.63 (0.96-2.71) | 0.058 |
| Clinical N Status (N-positive *vs* N-negative) | 1.22 (0.62-2.4) | 0.574 |  | 1.2 (0.56-2.56) | 0.634 |
| Clinical TNM Stage (III/IV *vs* II) | 3.72 (0.6-22.97) | 0.158 |  | 3.75 (0.5-28.06) | 0.198 |
| CEA (< 5.0 ng/mL *vs* ≥ 5.0 ng/mL) | 0.81 (0.48-1.37) | 0.438 |  | 0.86 (0.5-1.47) | 0.583 |
| CA19-9 (< 37 U/mL *vs* ≥ 37 U/mL) | 0.89 (0.46-1.71) | 0.72 |  | 0.97 (0.5-1.89) | 0.934 |
| CA72-4 (< 6.7 μg/L *vs* ≥ 6.7 μg/L) | 0.79 (0.54-1.15) | 0.216 |  | 0.77 (0.53-1.13) | 0.179 |
| Neurological Invasion (Yes *vs* No) | 1.21 (0.78-1.87) | 0.386 |  | 1.38 (0.89-2.13) | 0.151 |
| Vascular Invasion (Yes *vs* No) | 1.49 (1-2.21) | ***0.048**** |  | 1.88 (1.27-2.78) | ***0.002**** |
| ypT Stage (T3/4 *vs* T0/1/2) | 1.54 (1.05-2.25) | ***0.027**** |  | 1.67 (1.14-2.45) | ***0.008**** |
| ypN Stage (N2/3 *vs* N0/1) | 1.56 (1.03-2.37) | ***0.038**** |  | 1.74 (1.14-2.65) | ***0.011**** |
| Newly detected distant metastasis during or after NAC (Yes *vs* No) | 5.5 (2.07-14.59) | ***0.001**** |  | 9.09 (4.27-19.37) | ***< 0.001**** |
| ypTNM Stage (III/IV *vs* 0/I/II) | 1.58 (1.08-2.32) | ***0.018**** |  | 1.79 (1.23-2.62) | ***0.003**** |
| CAP Degree (2/3 *vs* 0/1) | 1.83 (1.18-2.84) | ***0.007**** |  | 2.23 (1.44-3.46) | ***< 0.001**** |

NAC: neoadjuvant chemotherapy; yp: postoperative pathological stage after neoadjuvant chemotherapy; CAP: the tumor regression grading (TRG) system proposed by the College of American Pathologists; HR: hazard ratio; CI: confidence interval; ** P < 0.05*
