# Supplementary material for: Serum GDF15 as a potential biomarker for treatment response and survival in patients with resectable locally advanced gastric cancer undergoing neoadjuvant chemotherapy
Source: Front Mol Biosci. 2026 Oct 7;13:1948454. doi: 10.3389/fmolb.2026.1948454 (PMC13683077; doi:10.3389/fmolb.2026.1948454)
Supplement: Supplementary file 4 [file Table4.DOCX]

Table 4. Multivariable Cox regression analysis of factors associated with survival of patients

| **Variables** | **OS** | |  | **DFS** | |
| --- | --- | --- | --- | --- | --- |
|  | **HR (95%CI)** | **P-value** |  | **HR (95%CI)** | **P-value** |
| Serum GDF15 concentration, log10-transformed (per 1-unit increase) | 2.09 (1.12-3.92) | ***0.021**** |  | 1.95 (1.02-3.51) | ***0.048**** |
| Age (< 60 *vs* ≥ 60) | 0.61 (0.41-0.91) | ***0.016**** |  | 0.64 (0.43-0.95) | ***0.027**** |
| Vascular Invasion (Yes *vs* No) | 1.33 (0.88-2.01) | 0.174 |  | 1.59 (1.05-2.41) | ***0.029**** |
| CAP Degree (2/3 *vs* 0/1) | 1.71(1.08-2.7) | ***0.022**** |  | 2.02 (1.27-3.2) | ***0.003**** |

CAP: the tumor regression grading (TRG) system proposed by the College of American Pathologists; HR: hazard ratio; CI: confidence interval; ** P < 0.05*
