# Supplementary material for: Serum GDF15 as a potential biomarker for treatment response and survival in patients with resectable locally advanced gastric cancer undergoing neoadjuvant chemotherapy
Source: Front Mol Biosci. 2026 Oct 7;13:1948454. doi: 10.3389/fmolb.2026.1948454 (PMC13683077; doi:10.3389/fmolb.2026.1948454)
Supplement: Supplementary file 5 [file Table5.DOCX]

Table S1. Standardized mean differences before and after propensity score matching

| **Variables** | | **SMD** | | **Balance status** | |
| --- | --- | --- | --- | --- | --- |
|  | **Before matching** | | **After matching** | **Before matching** | **After matching** |
| distance | 0.621448958 | | 0.053261380 | Imbalanced | Balanced |
| Sex_ | -0.189747022 | | -0.023821100 | Imbalanced | Balanced |
| Age | 0.364802529 | | -0.140083060 | Imbalanced | Imbalanced |
| NRS | -0.290885429 | | 0.093181424 | Imbalanced | Balanced |
| Tumor_antrum | -0.621300317 | | 0.000000000 | Imbalanced | Balanced |
| Tumor_body | -0.320193279 | | -0.093287760 | Imbalanced | Balanced |
| Tumor_cardia | 0.042919231 | | 0.088218050 | Balanced | Balanced |
| Differentiation_Moderate | -0.220945420 | | -0.172944620 | Imbalanced | Imbalanced |
| Differentiation_Poor | 0.269353745 | | 0.169244750 | Imbalanced | Imbalanced |
| Differentiation_Well | -0.111725412 | | -0.153605630 | Imbalanced | Imbalanced |
| Lauren_Diffuse | -0.019025181 | | 0.071687150 | Balanced | Balanced |
| Lauren_Intestinal | -0.014452829 | | -0.054690950 | Balanced | Balanced |
| Lauren_Mixed | 0.041280111 | | 0.019706590 | Balanced | Balanced |
| Signet | 0.195357112 | | -0.082897580 | Imbalanced | Balanced |
| Her2_0 | -0.005762914 | | 0.000000000 | Balanced | Balanced |
| Her2_1 | 0.033053756 | | 0.056973550 | Balanced | Balanced |
| Her2_2 | -0.131292922 | | -0.060868340 | Imbalanced | Balanced |
| Her2_3 | 0.147066127 | | 0.056688319 | Imbalanced | Balanced |
| NAC_FOLFOX6 | -0.124367372 | | -0.030323890 | Imbalanced | Balanced |
| NAC_SOX | 0.188460594 | | 0.019418390 | Imbalanced | Balanced |
| NAC_XELOX | -0.139677195 | | -0.038191840 | Imbalanced | Balanced |
| cT_2 | -0.236455825 | | -0.061516570 | Imbalanced | Balanced |
| cT_3 | 0.062729473 | | 0.073975680 | Balanced | Balanced |
| cT_4 | 0.138585990 | | 0.066510350 | Imbalanced | Balanced |
| cN | 0.222438422 | | 0.087081220 | Imbalanced | Balanced |

SMD: standardized mean difference; PSM: propensity score matching; *absolute SMD < 0.10 indicates adequate covariate balance.*
