# Supplementary material for: Serum GDF15 as a potential biomarker for treatment response and survival in patients with resectable locally advanced gastric cancer undergoing neoadjuvant chemotherapy
Source: Front Mol Biosci. 2026 Oct 7;13:1948454. doi: 10.3389/fmolb.2026.1948454 (PMC13683077; doi:10.3389/fmolb.2026.1948454)
Supplement: Supplementary file 6 [file Table6.DOCX]

**Figure 1:** **Flowchart of the study and serum GDF15 grouping.**

**(A)** Study flowchart. After application of the eligibility criteria, 318 patients underwent baseline serum GDF15 measurement. The median concentration in this unmatched cohort was used as the cutoff before PSM, resulting in 159 patients in each GDF15 group. Following 1:1 PSM, 128 patients were retained in each group. **(B)** Serum GDF15 concentrations in healthy controls and patients in the matched GDF15-L and GDF15-H groups. The groups were defined using the fixed pre-PSM median cutoff of 2147.3 pg/mL. *p < 0.05 and ***p < 0.001.

**Figure 2: Influence of GDF15 expression on overall survival and disease-free survival of resectable LAGC patients receiving NAC.**

The Kaplan-Meier curves show overall survival **(A)** and disease-free survival **(B)** of GDF15-H/L patients.

**Figure 3: Representative immunohistochemical (IHC) staining of GDF15 in** **pretreatment endoscopic biopsy specimens obtained before NAC.**

The IHC staining results illustrate the expression status of GDF15 in the tumor interior of patients in GDF15-H/L groups. The dotted lines show the magnified area. Scale bar, 100 μm.

**Figure 4: Representative abdominal enhanced CT images of patients.**

From left to right, the images of each row show the change of tumor size in GDF15-H/L patients before and after receiving NAC. The tumor region is marked by red dashed boxes.

**Figure 5: Prognostic nomograms to predict the OS and DFS of resectable LAGC patients receiving NAC.**

**(A)** Prognostic nomograms to predict the overall survival (OS). **(B)** Prognostic nomograms to predict the disease-free survival (DFS). CAP: the tumor regression grading (TRG) system proposed by the College of American Pathologists.

**Supplementary Figure S1: Quantitative real-time PCR validation of GDF15 mRNA expression in pretreatment endoscopic biopsy specimens.**

**(A)** Relative GDF15 mRNA expression in pretreatment frozen endoscopic biopsy specimens from the GDF15-L/CAP 0, GDF15-L/CAP 3, GDF15-H/CAP 0, and GDF15-H/CAP 3 subgroups (n = 6 per subgroup). GDF15 expression was normalized to HPRT1 and calculated using the 2^−ΔΔCt method. Bars represent the mean ± standard deviation, and each dot represents one patient. Differences were evaluated using two-way ANOVA with serum GDF15 group and CAP grade as the two factors, followed by Šídák-adjusted comparisons between CAP grades 0 and 3 within each serum GDF15 group. The uppermost bracket indicates the main effect of baseline serum GDF15 group. **(B)** Spearman correlation between baseline serum GDF15 concentration and relative tissue GDF15 mRNA expression across all 24 validation samples. The dashed line represents a least-squares linear fit for visualization only and was not used to calculate Spearman’s correlation coefficient. Spearman’s ρ = 0.90, P < 0.001. CAP grade 0 indicates the absence of residual viable tumor cells after NAC, whereas CAP grade 3 indicates minimal or no pathological tumor response. *P < 0.05, **P < 0.01, and *P < 0.001.

**Supplementary Figure S2. Love plot showing covariate balance before and after propensity score matching.**

Red circles and blue triangles represent the absolute standardized mean differences before and after matching, respectively. The vertical dashed line indicates the prespecified balance threshold of 0.10.
